# The Representation of Finger Movement and Force in Human Motor and Premotor Cortices

**DOI:** 10.1523/ENEURO.0063-20.2020

**Published:** 2020-08-14

**Authors:** Robert D. Flint, Matthew C. Tate, Kejun Li, Jessica W. Templer, Joshua M. Rosenow, Chethan Pandarinath, Marc W. Slutzky

**Affiliations:** 1 Department of Neurology, Northwestern University, Chicago, IL 60611; 2 Shirley Ryan AbilityLab, Chicago, IL 60611; 3 Department of Neurological Surgery, Northwestern University, Chicago, IL 60611; 4 Computation and Neural Systems Program, California Institute of Technology, Pasadena, CA 91125; 5 Department of Physical Medicine and Rehabilitation, Northwestern University, Chicago, IL 60611; 6 Department of Biomedical Engineering, Emory University and Georgia Institute of Technology, Atlanta, GA 30322; 7 Department of Neurosurgery, Emory University, Atlanta, GA 30322; 8 Emory Neuromodulation and Technology Innovation Center (ENTICe), Emory University, Atlanta, GA 30322; 9 Department of Physiology, Northwestern University, Chicago, IL 60611; 10 Department of Biomedical Engineering, Northwestern University, Chicago, IL 60611

**Keywords:** cortex, electrocorticography, grasp, human, kinematic, kinetic

## Abstract

The ability to grasp and manipulate objects requires controlling both finger movement kinematics and isometric force in rapid succession. Previous work suggests that these behavioral modes are controlled separately, but it is unknown whether the cerebral cortex represents them differently. Here, we asked the question of how movement and force were represented cortically, when executed sequentially with the same finger. We recorded high-density electrocorticography (ECoG) from the motor and premotor cortices of seven human subjects performing a movement-force motor task. We decoded finger movement [0.7 ± 0.3 fractional variance accounted for (FVAF)] and force (0.7 ± 0.2 FVAF) with high accuracy, yet found different spatial representations. In addition, we used a state-of-the-art deep learning method to uncover smooth, repeatable trajectories through ECoG state space during the movement-force task. We also summarized ECoG across trials and participants by developing a new metric, the neural vector angle (NVA). Thus, state-space techniques can help to investigate broad cortical networks. Finally, we were able to classify the behavioral mode from neural signals with high accuracy (90 ± 6%). Thus, finger movement and force appear to have distinct representations in motor/premotor cortices. These results inform our understanding of the neural control of movement, as well as the design of grasp brain-machine interfaces (BMIs).

## Significance Statement

The human ability to manipulate objects is central to our daily lives and requires control of both grasping movement and force. Here, we explored how these motor activities are represented at the level of the cortex. Understanding these representations will influence the design of brain-machine interfaces (BMIs) to restore function after paralysis. We recorded electrocorticography (ECoG) from seven human subjects who performed a sequential movement-force motor task. We found differences between the cortical representations of movement and force using decoding methods, deep learning, and a new neural ensemble metric. Thus, ECoG could be used in a BMI to control both movement and force behaviors. These results can potentially accelerate the translation of BMIs for individuals with paralysis.

## Introduction

The human ability to grasp and manipulate objects is central to our evolutionary success as tool users. The loss of this ability has a profound negative impact on overall quality of life. We rely in particular on our ability to precisely regulate movement and force, to close our fingers around an object, then exert isometric force sufficient to prevent slippage without crushing it. However, the neural origin of this process is not yet clear. In the current study, we sought to identify how movement and force are encoded at the cortical level when both are performed sequentially.

There is longstanding evidence for cortical representations of both movement (Moran and Schwartz, 1999) and force (Evarts, 1968). There is also indirect evidence that distinct neural control states are used for kinematics (movement) and kinetics (force). For example, motor learning of kinematics and kinetics in reaching occur independently of each other (Flanagan et al., 1999). Kinematic and kinetic control can be disrupted independently (Chib et al., 2009), and their errors can be separated during adaptation (Danion et al., 2013). Perhaps most relevant, Venkadesan and Valero-Cuevas (2008) found that electromyogram (EMG) activity patterns transitioned between separate, incompatible states during a one-finger, sequential movement-force task. Importantly, these transitions occurred before the fingertip’s contact with a surface, implying that changing neural states may “prepare” finger muscle activations for their upcoming role in regulating force. Here, we hypothesized that the transition between movement and force is encoded in motor and premotor cortical networks.

The specifics of cortical movement and force encoding are also relevant to brain-machine interface (BMI) design (Downey et al., 2018; Branco et al., 2019a; Slutzky, 2019; Rastogi et al., 2020). Restoration of hand grasp functionality is a high priority for individuals with paralysis (Blabe et al., 2015). Currently, BMIs using motor cortical signals control robotic or prosthetic hands (Hochberg et al., 2012; Yanagisawa et al., 2012; Wodlinger et al., 2015; Hotson et al., 2016) or functional electrical stimulation of paralyzed limbs (Pfurtscheller et al., 2003; Bouton et al., 2016; Ajiboye et al., 2017). However, most BMIs that have decoded grasp intent have focused on decoding kinematics of grasp aperture. One exception improved BMI-prosthetic hand control by scaling the neuronal firing rates (Downey et al., 2017) but did not examine the movement-force transition. Here, we hypothesized that force and kinematics of the hand are governed by different neural states in cortex.

In the current study, we used a sequential movement-force task to investigate changes in human cortical activity during transitions in behavioral mode: from premovement (preparation) to movement to force. We recorded subdural surface potentials [electrocorticography (ECoG)], finger kinematics, and applied force. We used ECoG spectral modulations to measure changes in the spatial patterns of movement-based and force-based decoding and to classify the behavioral mode of the subject. We found evidence of distinct movement and force encoding.

Recent work has characterized changes in cortical network activity during kinematic tasks as the temporal evolution of a dynamical system (Churchland et al., 2012; Pandarinath et al., 2018). Here, we examined whether neural state space changes accompanied behavioral mode transitions (from premovement to movement to force). We used latent factor analysis via dynamical systems (LFADS), a deep-learning method that uses sequential autoencoders to uncover trajectories in a low-dimensional neural state space from high-dimensional neural data (Pandarinath et al., 2018). We also calculated changes in a neural vector angle (NVA), obtained by treating the spectral features as elements of a high-dimensional neural vector. Both approaches showed that activity across a broad area of motor and premotor cortices exhibited tightly clustered trajectories through neural state space that were time-locked to the behavior. The NVA enabled us to average responses across subjects and create a generalized temporal profile of neural state space activity during the movement and force modes of human grasp. Together, these analyses indicate that distinct cortical states correspond to the distinct movement and force modes of grasp.

## Materials and Methods

### Subjects and recordings

Seven human subjects participated in the study (all male; ages 26–60, ordered chronologically). Six of the subjects required awake intraoperative mapping before resection of low-grade gliomas. Their tumors were located remotely to the cortical areas related to hand grasp, and no upper extremity sensorimotor deficits were observed in neurologic testing. Subject S6 underwent extraoperative intracranial monitoring before resection surgery for treatment of medication-refractory epilepsy. All human subjects were recruited at Northwestern University. The experiments were performed under protocols approved by the institutional review board. All subjects gave written informed consent before participating in the study. Subjects were recruited for the study if the site of their craniotomy, or their monitoring array was expected to include coverage of primary motor cortex.

**Figure 1.**
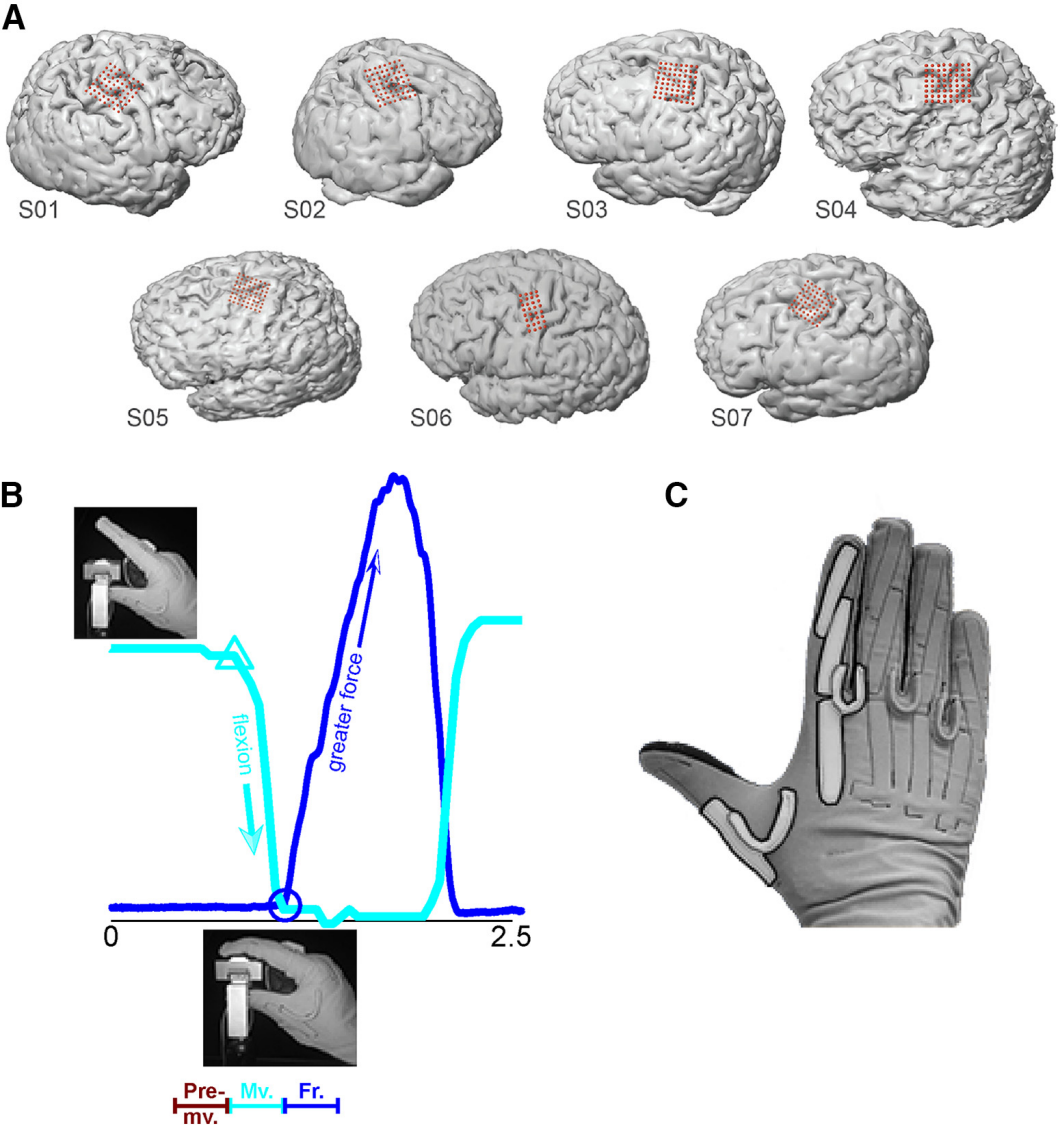
ECoG array placement, experimental task, and behavioral data. ***A***, In S1 through S5 and S7, we targeted the primary motor and premotor cortices. Array placement for S6 was determined by clinical need. For S1 and S2, we recorded ECoG from the right hemisphere; the other subjects’ ECoG were recorded from the left hemisphere. ***B***, One trial (∼2.5 s) of the kinematic-kinetic task. At the beginning of the trial, the subjects held their index finger in a neutral position (upper left photograph) until visually cued on a screen. Cyan trace, finger kinematics (amount of flexion; arbitrary units) during the trial. Cyan triangle, time of flexion movement onset. Upon contact with the force sensor (lower inset photograph), the subjects exerted isometric force until matching a force target on the screen with a cursor (data not shown). Blue trace, recorded force. Blue circle, time of force onset. At bottom is a schematic representation of behavioral mode segmentation, premovement (from target presentation until the start of flexion), movement (start of flexion until start of force), and force (from force onset lasting 500 ms). ***C***, We measured index finger flexion using a CyberGlove; movement onset was identified using the first PC calculated on the data from the highlighted sensors.

In all subjects except S6, we used 64-electrode (8 × 8) high-density ECoG arrays, with 1.5-mm exposed recording site diameter and 4-mm interelectrode spacing (Integra). Arrays were placed over hand motor areas, which we defined by: (1) anatomic landmarks, e.g., “hand knob” in primary motor cortex; (2) preoperative fMRI or transcranial magnetic stimulation to identify functional motor areas; and (3) direct electrocortical stimulation mapping. Intraoperative recordings took place after direct stimulation mapping. Intraoperative MRI navigation was performed with Curve (BrainLab). The recording arrays covered primary motor cortex, premotor cortex, and usually part of primary somatosensory cortex as well (Fig. 1*A*). In S6, electrode placement was determined by clinical need. For this subject, we used a 32-electrode (8 × 4) array with the same electrode size and spacing as our 64-electrode arrays.

We sampled ECoG at 2 kHz using a Neuroport Neural Signal Processor (Blackrock Microsystems). Signals were bandpass filtered between 0.3 and 500 Hz before sampling. Finger kinematics were recorded using a 22-sensor CyberGlove (Immersion). We recorded force with a custom-built load cell sensor. Kinematic and kinetic data were both sampled at the same rate as ECoG.

### Experimental protocol

The subjects executed repeated trials of a one-finger task that required isotonic movement and isometric force in sequence (Fig. 1*B*). At the beginning of each trial, the subjects were instructed to hold their index finger in a neutral posture (the “premovement” behavioral mode). After a cue, they executed a self-paced flexion movement, which brought the palmar surface of the index finger into contact with the force sensor. Upon contact, subjects were instructed to apply force to the sensor, thereby controlling a cursor on a monitor. Their task was to match the cursor’s vertical position to that of a force target presented on the monitor. Target force levels varied randomly from trial to trial (random-target pursuit task). Following a successful match (or a timeout of 2 s), the trial was complete, and the subject extended their finger back to the baseline (neutral) position. The next trial began after a delay of 1 s. Target presentation and cursor feedback were conducted by the open-source BCI2000 software (Schalk et al., 2004). The time resolution for both kinematic data acquisition and force cursor control was 50 ms.

Our task was designed to elicit movement by, and force using one finger, keeping the other fingers motionless in a flexed position. Therefore, our kinematic data consisted of the CyberGlove sensors that measured the motion of the index finger (Fig. 1*C*, highlighted). Dominant kinematic features were extracted via principal component analysis (PCA). We performed PCA only on data from the highlighted sensors in Figure 1*C*, retaining the first component to identify movement onset (the cyan trace in Figure 1*B* shows an example of the movement signal we used).

### Feature extraction

For all analyses, we extracted spectral features from each ECoG electrode. Here, each feature was the mean spectral power in a frequency band of interest. The sampling rate was 2000 Hz. To compute spectral power, we applied a Hanning window function to 256-ms segments of data, followed by a Fourier transform. We normalized the log of this power by subtracting the log of the mean power over the entire file, then extracted spectral features by averaging within frequency bands of interest (see next paragraph). The resolution of the frequency axis was 3.9 Hz. Each data segment (or time bin, to borrow nomenclature from past single-neuron studies) overlapped the previous by 231 ms, giving the analysis an effective temporal resolution of 25 ms.

We identified the feature boundaries (frequency bands of interest) by computing the event-related spectral perturbation (ERSP) for each electrode around the time of force onset. We then averaged the ERSPs for all electrodes in our dataset, and identified the frequency bands of interest: broadband low frequency (8–55 Hz) and broadband high frequency (70–150 Hz). Subsequent analyses were performed on the feature matrix for each subject. Each feature matrix was size NxM, where N is the number of time bins in the record, and M is 2*(number of electrodes)*10, where 10 was the number of time bins into the past (causal bins only).

### Population decoding of continuous movement and force

We decoded continuous movement kinematics and continuous isometric force, using all (non-noisy) electrodes from PM and M1 in each subject. For continuous decoding, the feature matrix served as input to a Wiener cascade decoder (Hunter and Korenberg, 1986). In the Wiener cascade, the output of a linear Wiener filter is convolved with a static nonlinearity (here, a third-order polynomial). We employed ridge regression to reduce the likelihood of overfitting because of the large feature space, as in Suminski et al. (2010). We evaluated decoding accuracy using the fractional variance accounted for (FVAF). We employed 11-fold cross-validation, using 9-folds for training, 1-fold for parameter validation (e.g., optimizing the free parameter in the ridge regression; Fagg et al., 2009), and 1-fold for testing. We report the median ± interquartile range (IQR) of FVAF across test folds. Movement and force were treated as separate, independent sources of information for continuous decoding. All sampled times were used to decode movement, whether the subject was in premovement, movement, force, or between trials. Likewise, all sampled times were used to decode force. The purpose of decoding continuous movement and force was to validate the information content of the ECoG signals. Thus, a high FVAF indicated that the ECoG signals encode information about times of active behavior (movement or force) as well as rest periods, and transitions among behavioral modes.

### Spatial mapping of decoding performance

We quantified the difference in the spatial representations of movement and force using two measures: (1) change in location of the peak single-electrode decoding performance, and (2) change in the overall spatial distribution of single-electrode decoding performance. For both analyses, we decoded continuous movement for each individual ECoG electrode using Wiener cascade decoders, as in the previous section. As in the previous paragraph, all data (regardless of behavioral mode) were used to evaluate decoding accuracy using the cross-validated FVAF. The spatial distribution of single-electrode movement decoding performance formed a “map” for the array. In a similar manner, we constructed a map of force decoding performance. We then analyzed these maps to reveal differences between movement and force spatial representation patterns on the cortical surface.

We compared the location of the overall peak of each decoding map for movement to that of force within each cross-validation fold. We report the absolute displacement between the peak performance location from force decoding versus that from movement decoding. Peak performance displacement quantifies the shift in location between movement and force in units of distance (here, in millimeters).

In addition, we compared the overall decoding map patterns. The map for a single fold can be treated as an image, with FVAF values corresponding to pixel intensities. We measured similarity among maps using a differencing metric common to image processing (Euclidean distance). We calculated the distance (D) between pairs of maps for individual folds. For example, a value of D_intra,3–4(force)_ = 0, where D is the difference metric, would indicate that the force decoding maps in folds 3 and 4 were identical. We compared the intermap distances across behavioral modes (movement vs force, D_inter_) to find the average decoding map difference between movement and force encoding on the cortex. We compared these to within-modality distances (D_intra(force)_, D_intra(mvt)_), which vary only because of time. That is, D_intra_ measured map differences within a behavioral mode, which can be attributed to variance in task performance across trials. Thus, D_intra_ values served as controls for D_inter_, which measured the map differences attributable to behavioral mode (movement or force). When calculating these distance metrics between performance maps, we scaled by the maximum possible distance between the maps, so that both D_inter_ and D_intra_ ranged from 0 to 1.

### LFADS

We used a deep learning algorithm known as LFADS to denoise ECoG features (Sussillo et al., 2016; Pandarinath et al., 2018). LFADS denoises neural activity based on the assumption that the observed patterns of neural modulation can be described as noisy observations of an underlying low-dimensional dynamical system. LFADS aims to extract a set of low-dimensional latent factors that describe neural population activity on a single-trial basis. When previously applied to spiking activity from populations of neurons, LFADS modeled observed spikes for each neuron as samples from an inhomogeneous Poisson process (called the firing rate), and attempted to infer this underlying firing rate for each neuron. In this study, since the ECoG features are continuous rather than discrete variables, the underlying distribution was taken to be Gaussian instead of Poisson. Specifically, the data were preprocessed by *z*-scoring each spectral feature. Then, the data were modeled following the equations in Sussillo et al. (2016), with the key modifications that:

(1) $$\mu_{r,t}={\text{W}}^{fac1}({\text{f}}_{t}),$$

(2) $$\sigma_{r,t}={\text{W}}^{fac2}({\text{f}}_{t}),$$

(3) $$x_{t}∼N(\mu_{r,t},\sigma_{r,t}^{2}),$$

where **x***_t_* represents the vector of *z*-scored spectral features at each timestep, and **f***_t_* represents the latent factors output by the LFADS recurrent neural network. For a given spectral feature r, $\mu_{r,t}$, and $\sigma_{r,t}$ represent the inferred time-varying mean and variance, respectively, for the *z*-scored spectral feature at each time step. $W^{fac1}$ and $W^{fac2}$ are matrices that map the latent factors onto $\mu_{r,t}$ and $\sigma_{r,t}$, respectively. These matrices have fixed weights across all time points. For each subject, the number of latent factors allowed was approximately half the total number of ECoG channels used. After applying LFADS, we used PCA to produce low-dimensional visualizations of the denoised ECoG features.

### NVA

To compactly represent the overall response of a subject’s feature set, we computed NVAs for each trial. This quantity is similar to the “muscle coordination pattern” angle of Venkadesan and Valero-Cuevas (2008). We selected features to include in the NVA calculations using the following method: first, we averaged the ECoG spectral intensity across trials, aligned to force onset. We then used unsupervised k-means clustering (three clusters) to partition the trial-averaged spectral power from the complete set of features. All M1/PM features served as inputs to the clustering algorithm. We evaluated this algorithm with two to five input clusters in each subject, using silhouette values to judge the quality of clustering. Grouping the features into three clusters produced the best groupings (with zero negative silhouette values in most subjects). Of the three output clusters, we selected the two that were well modulated with movement and/or force: a cluster of low-frequency features and a cluster of high-frequency features. These groupings for well-modulated features (low and high frequency) emerged natively from the unsupervised procedure, typically leaving one additional cluster of poorly-modulated features. Clustering was used only as a means of selecting ECoG features to include in NVA computations.

We calculated the NVA separately for the low-frequency and high-frequency features, as follows: a cluster of features with n members can be represented at time t as **m**(t) = [f_1_,f_2_,…,f_n_], where f is the value of an individual feature. We smoothed **m**(t) over five time bins (total 125 ms), then calculated the NVA:

(4) $$\theta (t)=\cos^{-1}\left(\frac{\text{m(}t\text{)}•{\text{m}}^{ref}}{\left\|\text{m(}t\text{)}\right\|\left\|{\text{m}}^{ref}\right\|}\right),$$

where **m** ^ref^ is the average value of **m**(t) over the 250-ms period before the time of maximum force exertion in the trial. We computed the NVA at each time bin over trials in each of the emergent clusters (low-frequency and high-frequency modulating), for each subject. Since the NVA transformed the data from feature values to a common coordinate system (angle between vectors, in degrees), it enabled us to average this quantity across subjects. To quantify differences in NVA values because of behavioral mode, we used the Kruskal–Wallis test of unequal medians on NVAs during “premovement,” “movement,” and “force” modes (illustrated in Fig. 1*B*). See also the following section for details of the behavioral mode labeling procedure.

### Discrete classification of behavioral mode

Our classification of behavioral mode used the same frequency-based features as we used in our continuous decoding analysis. Here, the data were selected and labeled as follows: time bins from target presentation to the start of finger flexion were labeled as premovement; time bins from the start of flexion to contact with the force sensor were labeled movement; time bins beginning at contact with the force sensor, continuing for 0.5 s were labeled force. An example of this behavioral mode labeling for a single trial of data are shown in Figure 1*B*. We limited the length of the force window to obtain more balanced class sizes. Data outside of the described time windows were discarded. The data were classified using two methods: support vector machines (SVMs) and boosted aggregate (bagged) trees. The classification analyses used 5-fold cross-validation. Within each fold, we trained on (or tested) every individual 25-ms time bin. The reported accuracy measures are the median ± IQR of correctly classified time bins across all test folds. Because the class sizes were not exactly equal, the chance level performance of the 3-class classifier was not necessarily 1/3. We calculated the true chance level performance by shuffling the class labels and then repeating the analysis. We repeated this procedure 1000 times for each recording.

### Experimental design and statistical analysis

We conducted the experiments and analyzed the data using a within-subject design. We used non-parametric statistics to report continuous kinematics and continuous force decoding accuracy, as the decoding accuracy values (FVAF) were distributed non-normally across cross-validation folds. To compare maps of decoding performance, we conducted a one-tailed Wilcoxon signed-rank test, with Bonferroni correction for multiple comparisons. Differences in NVA during behavioral modes were tested using a Kruskal–Wallis test. For the discrete decoding of behavioral mode, we also used a Kruskal–Wallis test to identify statistical differences between ECoG feature-based decoding and LFADS-cleaned feature decoding.

## Results

We recorded ECoG from seven human subjects with brain tumors or epilepsy who required intraoperative or extraoperative mapping as part of their clinical treatment. In all subjects, ECoG coverage included at least part of primary motor and premotor cortices (Brodmann areas 4 and 6). In some cases, coverage also included prefrontal and/or postcentral cortices (Fig. 1*A*). However, we restricted our analyses to electrodes covering primary motor and premotor cortices. The subjects performed a cued one-finger task requiring an isotonic flexion movement, followed by isometric flexion to specified force targets. Movement and isometric flexion were performed sequentially (Fig. 1*B*). This task was adapted from Venkadesan and Valero-Cuevas (2008). We recorded the finger joint kinematics (based on the sensors highlighted in Fig. 1*C*) as well as the force generated by isometric flexion.

### ECoG feature modulations were time-locked with movement and force

Following Collard et al. (2016), we used event-aligned plots to visualize event-related changes in ECoG spectral features, specifically to understand how tightly these features modulated with behavioral events. We examined modulation with respect to (1) the start of finger flexion movement and (2) the start of isometric force exertion. For each feature, we constructed an “intensity raster” by windowing the feature’s data, then plotting as trial number versus perievent time. We sorted trials by the elapsed time between events.

**Figure 2.**
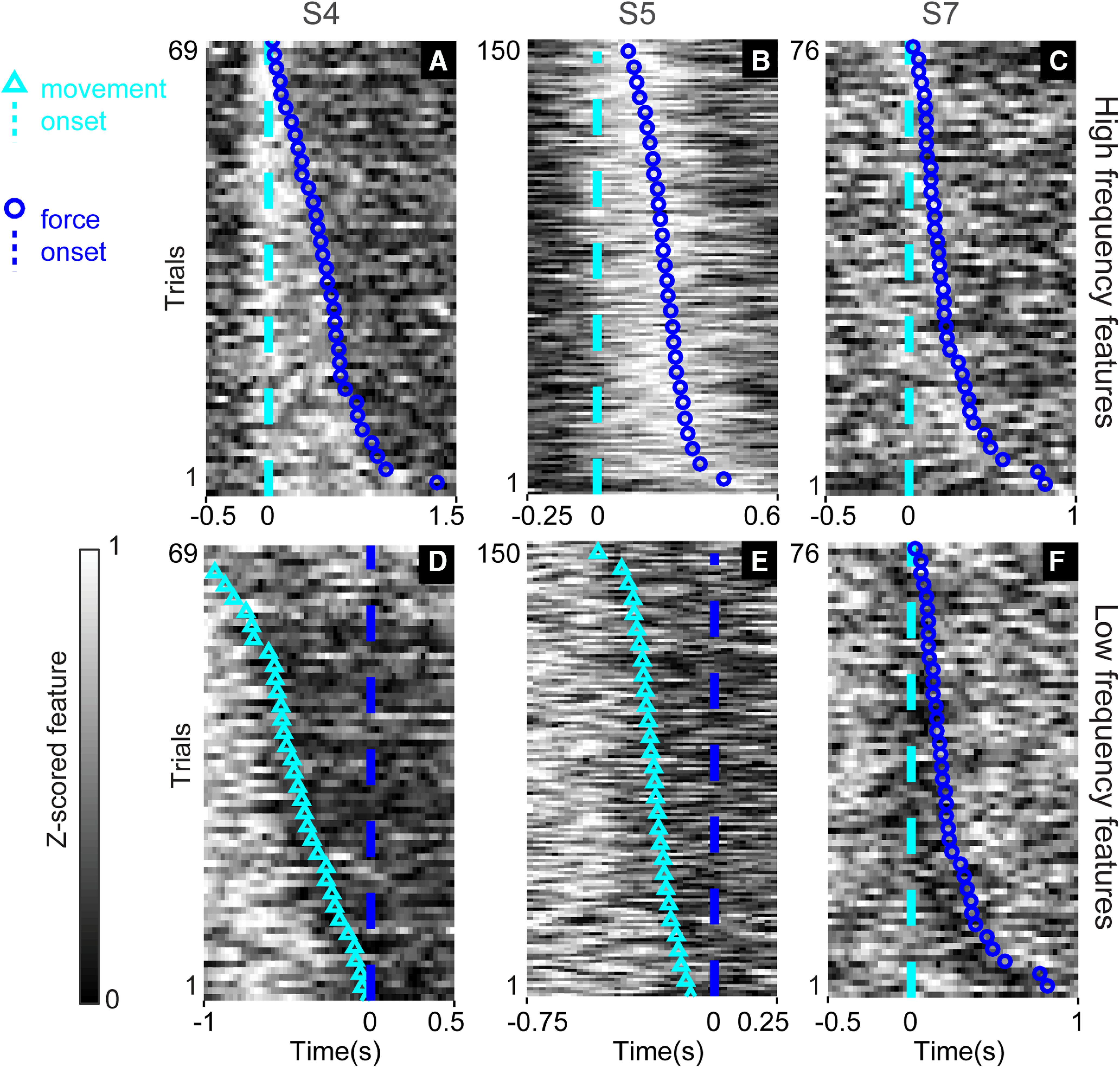
Spectral power modulation during the movement-force grasp task. Each panel shows data from a high-frequency or low-frequency spectral feature taken from an individual ECoG electrode. The single-trial frequency band power (grayscale in each plot) was *z*-scored and aligned either to movement onset (cyan dashed lines; ***A****–****C***, ***F***) or to force onset (blue dashed lines; ***D***, ***E***). Blue circles show force onset times when trials were aligned to movement onset. Cyan triangles show movement onset times when trials were aligned to force onset. High-frequency features (***A–C***) exhibited power increases, which could be time locked to both movement and force (***A***) or force only (***B***, ***C***). Low-frequency features (***D–F***) exhibited power decreases just preceding, and aligned to, the onset of movement (***D***, ***E***), or aligned to the start of force (***F***).

We constructed intensity raster plots for each feature in our dataset (two features per non-noise electrode, 722 total features in the dataset). Overall, we found a diverse set of activity patterns during movement and force production. Figure 2*A* shows an example of a high-frequency feature that appears to encode both movement and force, showing increased activity at the transition from premovement to movement (Fig. 2*A*, left of dashed line) and decreased activity after force onset (right of blue circles). Some high-frequency feature modulations were time-locked only to force execution (Fig. 2*B*,*C*). Examples of low-frequency features exhibiting power decreases at movement onset are shown in Figure 2*D*,*E*. Low-frequency power decrease could also be time-locked to the start of force, instead (Fig. 2*F*). Note that Figure 2*B*,*E* shows high-frequency and low-frequency features from the same ECoG electrode, illustrating that two behavioral modes can be encoded differently by high-frequency and low-frequency information on the same electrode. Overall, the results exemplified in Figure 2 indicate a heterogeneous set of spectral feature responses to movement and force; in fact, we did not find a simple way to combine feature intensity data that completely summarized the individual features’ responses across high-frequency or low-frequency domains. Therefore, we also examined population-level measures to obtain a more generalized description of how M1/PM represents kinematic-kinetic behavior.

### Continuous movement and force were decoded with high accuracy using ECoG

We used a Wiener cascade approach to build multi-input, single-output models for decoding behavior. We built one such model to decode the continuous time course of finger movement kinematics using both high and low spectral features from all (M1/PM) electrodes. A separate model was built to decode continuous isometric force from the same electrodes. Both movement and force were decoded at all times (not only during active movement or active force) using a cross-validated design. The resulting decoding accuracy was high for both force and kinematics: the FVAF ranged from 0.4 ± 0.1 (median ± IQR) to 0.8 ± 0.1 for the individual subjects. Across subjects, the overall median FVAF was 0.7 ± 0.2 for force decoding and 0.7 ± 0.3 for movement decoding. Statistically, the null hypothesis that movement kinematics and force were decoded with equivalent accuracy could not be rejected (Kruskal–Wallis test, *p* = 0.6); thus, any differences between movement and force representations were not due simply to decoding one quantity better than the other.

### Spatial mapping of decoding performance shows different cortical representations of movement and force

**Figure 3.**
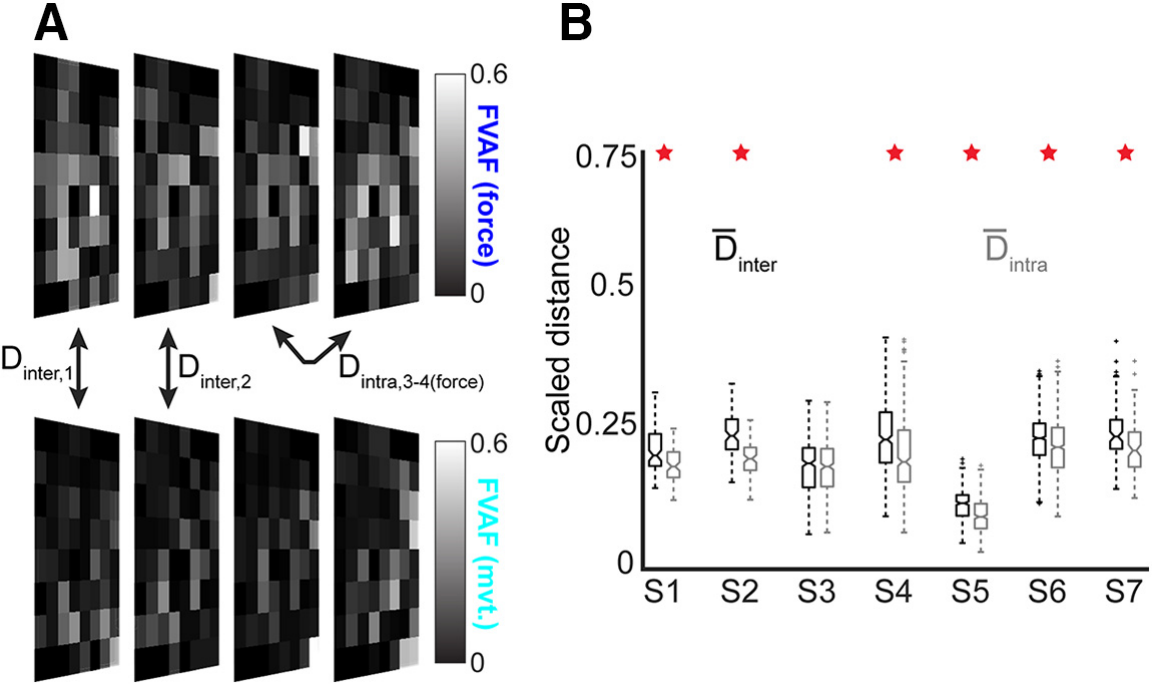
Decoding maps reveal changes in the cortical representations of movement and force. ***A***, Example decoding maps for S4; 4 folds of data are shown, the actual analysis used 10 folds per recording. Square recording arrays are shown in a rotated perspective for compact visualization. We compared single-electrode decoding maps for movement (top) and force (bottom) using a distance metric $D_{inter}$ for every possible combination of fold pairs. As a control, we calculated $D_{intra}$ between all possible fold pairs, for within-movement and within-force decoding. ***B***, Boxplot of distance measures for all subjects. The central horizontal line in each box shows the median, while the notches show 95% confidence intervals. Overall, the median $D_{inter}$ was significantly greater than the median $D_{intra}$ in six of seven subjects (red stars). Note that the maps in ***A*** show 64 channels; for the distance measures in ***B***, only the PM/M1 electrodes were included.

We next quantified the difference in the spatial representations of force and movement on the cortical surface, using two metrics: (1) change in location of the peak decoding performance electrode (Table 1), and (2) change in overall decoding map pattern (Fig. 3). A previous study found that decoding maps’ peak performance locations differed when two different fingers were used for an isometric force task (Flint et al., 2014). Here, we found that the peak performance location was different for movement and force decoding. The displacement (between movement and force) of the peak decoding performance ranged from 3.2 ± 5.4 to 16.5 ± 8.8 mm across subjects (mean ± SD over folds; Table 1). The mean (±SE) displacement of peak performance for all subjects was 9.9 ± 2.0 mm.

**Table 1:** Displacement of peak location (in mm) for movement decoding performance relative to force decoding performance in each subject

	Mean	±	SD
S1	16.1	±	4.1
S2	16.5	±	8.8
S3	3.2	±	5.4
S4	10.2	±	8.4
S5	4.2	±	6.6
S6	8.8	±	5.4
S7	10.7	±	8.0

To place these distances in context, a standard ECoG array for epilepsy use has an interelectrode distance of 10 mm, highlighting the advantages of using high-density ECoG arrays (the electrode arrays used here had an interelectrode distance of 4 mm). See also Wang et al. (2016).

In addition to changes in peak decoding location, there were differences between movement and force in their respective overall decoding map patterns (Fig. 3). The between-mode distance D_inter_, which measured differences between the movement-force maps (see Materials and Methods), was significantly greater than the within-mode distance D_intra_ in six of seven subjects (*p* < 3 × 10^−5^ except S3, where *p* = 0.19; one-tailed Wilcoxon signed-rank test with Bonferroni correction for multiple comparisons; see Fig. 3*B*). This indicates that the spatial distribution of decoding as a whole changed significantly between movement and force and that this change was greater than what would be expected from behavioral variation. Taken together, these results indicate that the spatial representations of movement and force on the cortical surface are different.

### Differences in premovement, movement, and force behavioral modes were reflected in a dynamical systems model of M1/PM network activity

**Figure 4.**
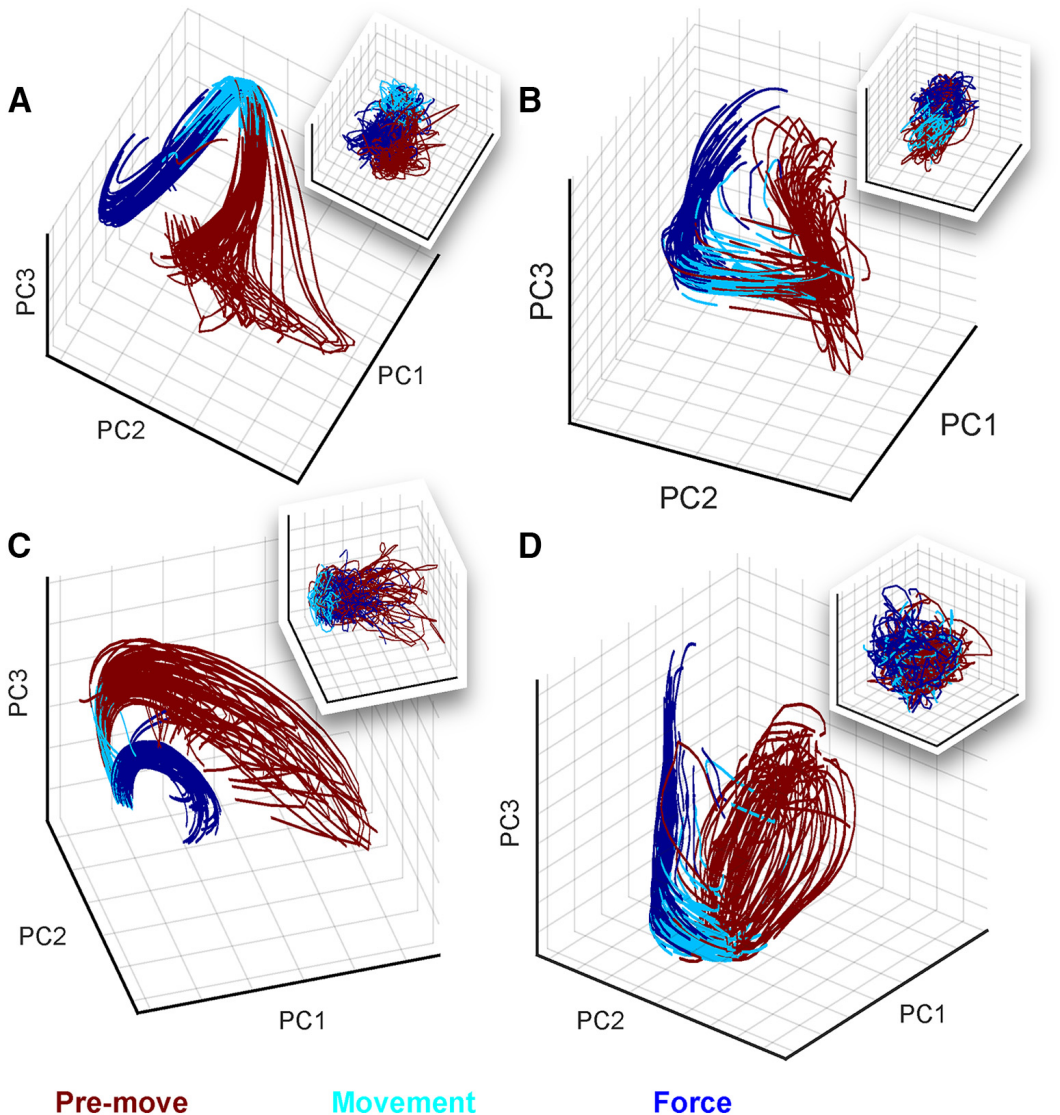
Modeling ECoG features as an underlying dynamical system using LFADS uncovers repeatable trajectories through a low-dimensional state space during the kinematic-kinetic task. Shown are LFADS-PCs (labeled as PC for simplicity) derived from high-frequency (***A***, ***B***) and low-frequency (***C***, ***D***) ECoG features. Single-trial trajectories are shown for subjects S5 (78 trials; ***A***, ***C***) and S6 (73 trials; ***B***, ***D***). Inset boxes in each panel show the trajectories resulting from PCA performed directly on the ECoG features (without LFADS). The color code at bottom defines the portion of each trial corresponding to each behavioral mode.

We next examined the activity of the recorded cortical network as a whole during the movement-force behavior. The preceding spectral/spatial analyses (Figs. 2, 3) treated individual ECoG electrodes as independent sources of information. Here, we instead sought a low-dimensional representation to clarify and summarize the activity of the cortical network during the time course of the behavior. We used LFADS (Pandarinath et al., 2018) to generate low-dimensional representations of single-trial activity in the ECoG feature state space (see Materials and Methods). To visually summarize the factors, we computed PCs of the LFADS-denoised ECoG features (labeled LFADS-PCs). Figure 4 shows the underlying dynamics for S5 and S6 during trials of the kinematic-kinetic task, color-coded by behavioral mode. At the start of the task (premovement), the high-frequency and low-frequency latent factors tended to be distributed through a relatively broad region of the state space (Fig. 4*A*, red). Before the start of movement, the latent factors tended to converge onto a smaller region of state space, and their trajectories through the movement (cyan) and force (blue) periods of the task were more tightly grouped. Moreover, each time period of the task occupied a different part of state space (note the grouping of colors in Fig. 4). To illustrate the impact of LFADS in revealing well-ordered, low-dimensional state space representations, we also performed PCA directly on the ECoG features (PCA-only; Fig. 4, inset boxes). In some cases, PCA-only resulted in a rough grouping of behavioral modes (premovement, movement, and force) in neural state space (Fig. 4*A*). However, the individual PCA-only trial trajectories remained highly variable, unlike the highly repeatable LFADS-PC trajectories. In other cases, PCA-only did not allow us to resolve a low-dimensional state space representation with identifiable groupings at all (Fig. 4*D*). Contrasting the LFADS-PC plots with the PCA-only plots (i.e., compare Fig. 4, each panel, inset) illustrates the benefit of LFADS in visualizing this dataset. We quantified this benefit in Table 2, which shows the number of components required to account for 90% of the variance in the data, with and without LFADS.

**Table 2:** Number of PCs required to account for 90% of the variance in the ECoG features (PCA-only) or the latent factors (LFADS PCs)

	PCA-only	LFADS PCs
S1	43/66	2/66
S2	32/48	2/48
S3.1	26/44	2/44
S3.2	24/32	3/32
S4.1	40/74	3/74
S4.2	35/72	2/72
S5.1	19/36	2/36
S5.2	24/40	2/40
S6.1	28/38	4/38
S6.2	27/36	3/36
S6.3	27/36	3/36
S7	32/78	2/78

### An NVA summarizes temporal changes across the feature space

Visualizing the low-dimensional state space with LFADS-PCs reinforced the idea that premovement, movement, and force behavioral modes are well-represented in neural state space. However, those methods did not allow us to generalize across subjects. Therefore, we used a second metric for summarizing the modulations of feature space across trials and subjects: the NVA. The NVA θ(t) is the angle at time t between a neural vector **m**(t) and its reference direction, **m** ^ref^ (see Materials and Methods). Here, the high-dimensional vector **m**(t) was comprised of M1/PM ECoG spectral features. The reference vector **m** ^ref^ was calculated during a window before the moment of peak force in each trial. Therefore, θ(t) measures the dissimilarity between the ECoG features at each moment with their values during peak force generation.

**Figure 5.**
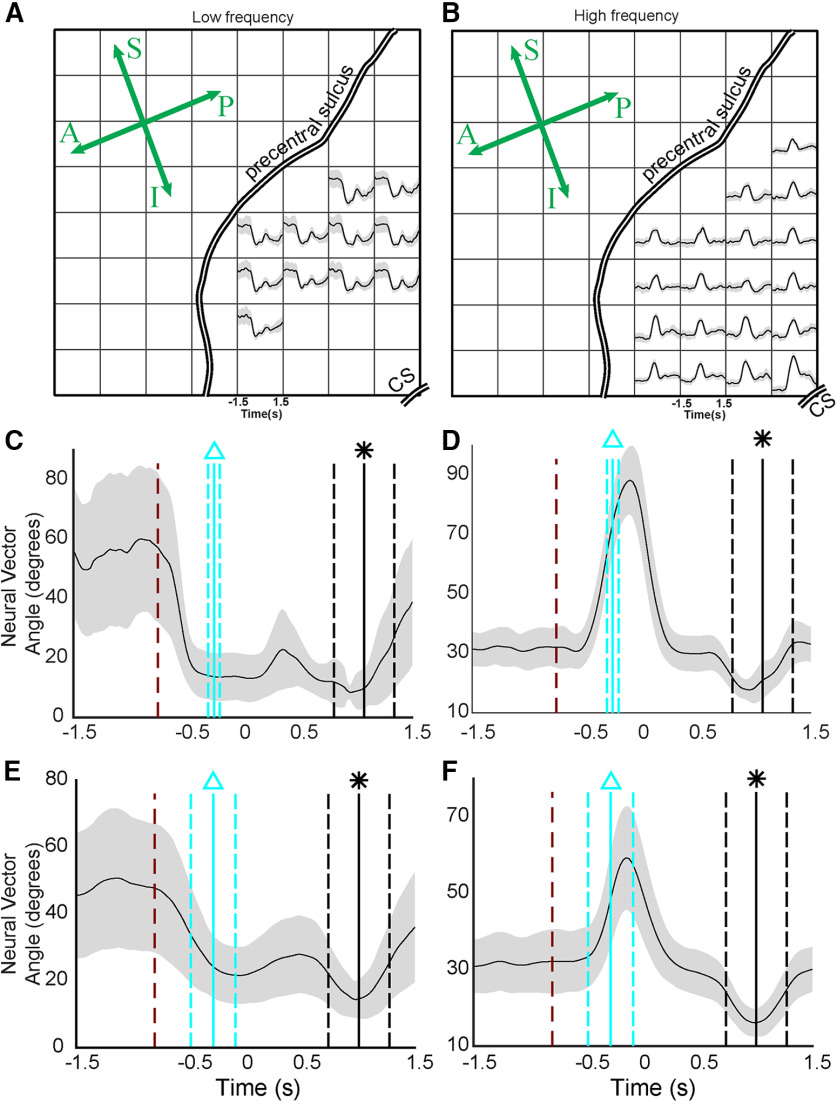
The NVA summarizes the cortical state change associated with the behavioral mode change from movement to force. ***A***, ***B***, Electrodes selected for S5, using k-means clustering. CS; central sulcus. Anterior-posterior and superior-inferior are indicated on the rosette; compare to Figure 1*A*. ***A***, ***B***, Two of the three resulting clusters; the unsupervised cluster analysis natively divided the responses into low-frequency and high-frequency responses. ***C***, The NVA, θ(t) for the low-frequency features selected in ***A***. The dark red dashed line shows the average time of target appearance, relative to force onset (time = 0). The vertical cyan lines show the mean (solid line) and standard deviation (dashed lines) of movement onset, relative to force onset. The vertical black lines show the time of maximum force for each trial (equivalent to the reference period **m** ^ref^). ***D***, The NVA for the high-frequency features shown in ***B***. ***E***, ***F***, NVAs calculated across all trials, all subjects in the study. Labeling conventions are the same as in ***C***, ***D***.

To maximize the signal-to-noise ratio of θ(t), the elements of **m**(t) were selected using a cluster analysis (see Materials and Methods). The resulting clusters were typically (1) a cluster of well-modulated low-frequency features (Fig. 5*A*), (2) a cluster of well-modulated high-frequency features (Fig. 5*B*), and (3) a cluster of poorly modulated features (data not shown). We computed θ(t) separately for clusters (1) and (2) in each subject (Fig. 5*C*,*D*). The NVA recasts feature modulations for each trial into a common unit (angular difference in degrees). Therefore, we were able to combine NVA results across all trials in all subjects, yielding a compact study-wide representation of the cortical response to the movement-force transition (Fig. 5*E*,*F*).

Across subjects, average low-frequency NVAs began to decrease immediately after the presentation of the cue to start the trial (Fig. 5*E*, red line) and reached their minimum value approximately at the start of flexion (Fig. 5*E*, cyan line). Accordingly, low-frequency NVA during movement was significantly lower than NVA during the premovement period (*p* < 10^−9^; Kruskal–Wallis test, Tukey HSD *post hoc* for all statistical comparisons in this section). By contrast, there was no significant difference between the movement period and force (*t* = 0 to *t* = 0.75) in the low-frequency NVAs (*p* = 0.32). High-frequency NVAs did not deviate from their premovement values at target presentation (Fig. 5*F*) instead changing just before the start of movement (Fig. 5*F*, cyan line). During movement, high-frequency NVAs were significantly higher than premovement NVA (*p* < 10^−9^), peaking just before the onset of force (Fig. 5*F*, approximately *t* = −130 ms relative to force onset). During the force behavioral mode, high-frequency NVA were overall lower than either movement (*p* < 10^−9^) or premovement (*p* < 10^−6^) periods.

Overall, the NVA provided a compact way to summarize cortical state space changes across subjects during the sequential movement-force task. Earlier, Figure 2 showed that responses of individual ECoG features could be quite heterogeneous in their modulations to behavioral events. Here, Figures 4, 5 showed that despite that heterogeneity of individual feature modulations, the information conveyed by populations of features exhibited repeatable, statistically significant patterns during these behaviors. Like Figure 2, the NVA results suggest the possibility of different cortical responses by particular parts of the frequency spectrum (low-frequency and high-frequency features). However, the NVA suggests that, while there may be exceptions (as seen in Fig. 2), this distinction may be a general characteristic of M1/PM cortices during the movement-force behavior.

### ECoG features enabled accurate classification of behavioral modes

**Figure 6.**
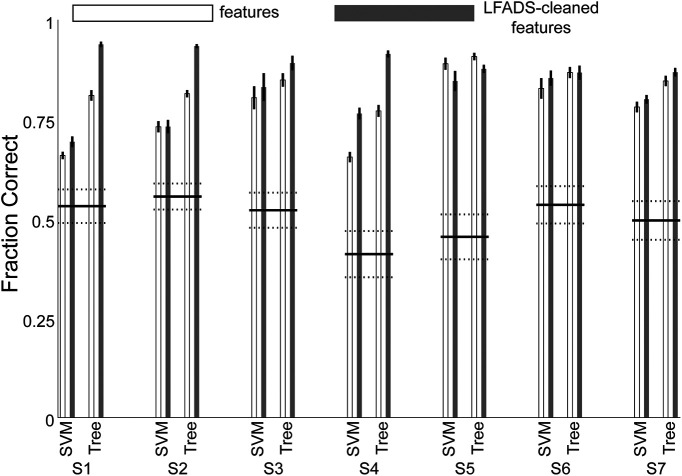
Decoding behavioral mode from ECoG features before and after LFADS denoising. The median classification accuracy was greater than chance for all subjects. Tree; boosted aggregate decision tree classifier.

Accurately decoding behavioral modes during grasp has potential applications for BMI design. For example, in response to evolving functional goals (e.g., changing from movement to force behavior when picking up an object), a BMI could switch control strategies. To estimate the accuracy such control might achieve, we tested whether the subjects’ behavioral modes could be decoded from cortical activity. We used the low-frequency and high-frequency ECoG spectral features to classify each time bin as one of three behavioral modes: premovement, movement, or force execution. The ground-truth behavior mode distinctions were labeled according to the movement onset and force onset events (for an example trial, see Fig. 1*B*). In parallel with the ECoG feature-based classification, we also classified behavioral mode using the LFADS-denoised features as inputs. This gave us a way to estimate the impact of cortical “noise” on the accuracy of decoding behavioral mode. We used two widely available classifiers: SVMs and boosted aggregate (bagged) decision trees. For each subject, we also calculated a chance decoding value (see Materials and Methods). We report classification accuracy for the two types of classifiers separately, evaluating both the features and the LFADS-denoised factors. The three behavioral modes were strongly differentiable in all subjects (Fig. 6). Overall, the tree-based classifier outperformed SVM, and LFADS-denoised features were decoded more accurately than the features without denoising (*p* = 1.9^−7^, Kruskal–Wallis test). For the tree-based classifier of LFADS-denoised features, median decoding accuracies for the subjects ranged from 87 ± 2% to 94 ± 1%, with an overall median value of 90 ± 6%, indicating that these three classes were highly separable. Statistically, the decoding accuracy for all subjects was significantly higher than chance. We emphasize here that each 25-ms time bin was decoded, rather than decoding behavioral modes as blocks of time. Thus, these behavioral modes have separable cortical representations on a 25-ms time scale.

## Discussion

Manipulating objects dexterously requires controlling both grasp kinematics and isometric force. Even simple activities like turning a doorknob, shaking hands, and lifting a cup of liquid could not be accomplished safely and quickly without both kinds of control. More than two decades ago, investigators began to appreciate that the central nervous system may handle these two vital aspects of motor behavior separately (Flanagan et al., 1999). Here, we found quantifiable differences in how the motor and premotor cortices represented behavioral mode, i.e., premovement, flexion movement, and isometric force. We found individual feature modulations that were time-locked to behaviorally relevant events, and could be observed on a single-trial basis (Fig. 2). As ensembles, the ECoG modulations constituted a neural state change, accompanying changes in behavioral mode. We were able to model this change using a dynamical systems approach (LFADS), and decode the subjects’ behavioral modes with high accuracy. Understanding neural state changes like these in the context of a functional grasp task will inform the design of dexterous grasp BMIs.

Generally, we achieved highly accurate decoding of the continuous time course of the behavioral variables (movement and force). These results compared favorably with prior studies decoding finger movement kinematics (Acharya et al., 2010; Nakanishi et al., 2014; Xie et al., 2018) and isometric force (Pistohl et al., 2013; Chen et al., 2014; Flint et al., 2014; Vaidya et al., 2019). Importantly, there was no significant difference in our ability to decode force and movement across subjects, implying that any differences in cortical representations of force and movement were not simply expressions of a superior decoding of one or the other.

Spatially, human cortical encoding of finger movement takes place over a widespread area (Schieber, 2002), including complex and overlapping representations of individual finger movements (Dechent and Frahm, 2003). ECoG recordings make it possible to examine cortical activity on these relatively large spatial scales (Slutzky and Flint, 2017). We found that the maps of decoding performance altered significantly across movement and force representations (across-mode) in six of seven subjects. We controlled for changes because of time or behavioral variability (within-mode), by comparing the between-mode maps to the within-mode maps. One potential explanation for the spatial map differences could be that the activating regions of the maps are simply shrinking during isometric force. Such an explanation is consistent with evidence pointing to less cortical modulation with isometric force than with movement (Hendrix et al., 2009). However, in this case we found that the peaks of the decoding maps changed location (Table 1), indicating that the maps shifted rather than merely growing or shrinking. These spatial decoding results are relevant to the design of BMIs, since any BMI that restores grasp should ideally execute both movement and force functions. There is evidence that representations of hand movements are preserved following amputation (Bruurmijn et al., 2017), although it remains to be shown whether the movement-force functional map change will remain in an individual with paralysis. Downey et al. (2017) found that applying a scaling factor to neuronal spike rates facilitated the ability of human BMI users to grasp objects with a prosthetic hand. The utility of such a scaling factor may be a reflection of the functional somatotopy of the cortex, although the current results suggest that amplitude scaling would not necessarily be the ideal method of accounting for the difference in movement and force representations. Here, we found the mean shift in peak decoding location was 9.9 mm, a sizeable distance in the cerebral cortex. The overall differences in spatial decoding maps (patterns of decoding), while significant, were not large. However, this was not unexpected for two related motor activities (movement and force, in the context of a grasp-like behavior) performed by the same finger.

Increasingly, spiking activity in small areas of motor cortex has been modeled as a dynamical system in an effort to parsimoniously describe and understand network-level neuronal activity. In this study, we used LFADS to uncover low-dimensional neural state spaces for each subject. LFADS-PCs were tightly grouped over trials and occupied distinct regions of state space during the premovement, movement, and force behavioral modes (Fig. 4). Both low-frequency and high-frequency LFADS-PCs were clearly separated in different behavioral modes. Some previous examples of modeling cortical dynamics using latent factors have analyzed single behavioral modes. For example, Vaidya et al. (2015) modeled both reach-related and grasp-related neural ensembles as linear dynamical systems to study learning. Also, Gallego et al. (2018) also showed that there were some differences in local M1 neuronal ensemble activity between kinematic and kinetic cursor control tasks. Our results show that dynamical systems modeling can elucidate the latent factors underlying a widespread cortical network in addition to local circuit networks. It was not surprising that latent factor state space trajectories evolved with time during each trial; indeed, this is a fundamental underlying assumption of the dynamical systems model. The significance of the LFADS-derived trajectories was their smooth, repeatable paths through distinct regions of state space during behavioral mode transitions. Compared with PCA-only state space trajectories, LFADS factors clustered more tightly and evolved much more repeatably in premovement, movement, and force behavioral modes.

We used the NVA to summarize spectro-temporal changes across electrodes and subjects. The average duration of high-frequency neural vector changes (∼300 ms; Fig. 5*F*) was substantially shorter than the average duration of the force-matching part of the behavioral task (∼1 s). A phasic rise in high γ modulation near the onset of behavior has been shown during other grasp force behaviors (Chen et al., 2014; Branco et al., 2019b), as well as isotonic movement (Flint et al., 2017). Single-neuron studies in nonhuman primates also support the phasic modulation with force onset (Hendrix et al., 2009), or more often, phasic-tonic modulation (Maier et al., 1993; Mason et al., 2002; Intveld et al., 2018). This agreement makes sense when considering that high-γ activity is often correlated with ensemble spiking. It appears that the onset of force behavior, or perhaps the transition from movement to force, is especially meaningful to the cortex when encoding grasp.

Our results support and extend the findings of Venkadesan and Valero-Cuevas (2008), who inferred from muscle activity that the human motor system uses two separate control strategies for movement and isometric force. Importantly, they observed muscle activity changing ∼100 ms before force onset, ruling out the conclusion that changes in EMG patterns are purely the result of the mechanical constraints of the behavior. In the current study, we chose **m** ^ref^ in part to facilitate comparison with that study. We found similarities between the cortical low-frequency NVA and their angular deviation for muscle coordination patterns (Fig. 2*A* from that study), although our low-frequency NVAs changed earlier: ∼350 ms before force onset, which is compatible with the delay between cortical and muscular activity. Changes in high γ activity patterns (reflected by the NVA), on the other hand, occurred around 130 ms before force onset. This time course of changing cortical activity is consistent with the earlier EMG results and with the concept that control strategies for movement and force are encoded in the motor and premotor cortices rather than subcortical systems. This argues against the hypothesis that differences in cortical activity during movement-force are due mainly to somatosensory feedback changes in the two states.

We believe the present data indicate that the cortical state spaces are different among premovement, movement, and force. One possible hypothesis to explain this difference is that additional muscles (other than index finger flexors) may have been recruited during force mode compared with movement mode, for example to additionally stabilize the wrist. While we were not able to include EMG recordings because of time and access limitations to our participants, recording EMG simultaneously with ECoG might allow us to test such a hypothesis. However, Venkadesan and Valero-Cuevas (2008), who recorded EMG (but not neural activity) in a similar task, found that neural control strategies changed within the scope of finger flexor muscles: that is, the same muscles were used in different recruitment patterns. Overall, these findings are also consistent with prior work showing that M1 neurons display muscle-like encoding (Oby et al., 2013).

We note that our behavioral task was chosen to recreate a naturalistic movement-force model of object grasp, and was not designed to systematically explore the finger-movement kinematic-kinetic space. Specifically, we note the caveat that movement behavior was not required to be as variable as force, since no explicit movement “targets” were designated (unlike force targets which varied randomly). Accordingly, we designed the analysis of spatial decoding map differences (Fig. 3) in such a way as to control for within-mode variation over time. In addition, we observed much larger differences between movement and force behavioral modes than within mode, in both the latent factor trajectories (Fig. 4) and in our statistical analysis of the NVA values (Fig. 5). Thus, the data still support distinct cortical modes that correspond to distinct behavioral modes.

Our decoding of the subjects’ time-varying behavioral mode has ramifications for BMI design, as demonstrated by Suminski et al. (2013). Suminski et al. addressed a longstanding limitation of BMIs: decoders trained on a given set of motor activities do not predict accurately outside those activities. Hierarchical BMIs, which include multiple decoders operating in parallel with a switching mechanism, may outperform those with a single decoder. In the context of hand function, a decoder trained only on movement data may not provide optimal control of a BMI for grasping and manipulating objects, either with a prosthetic hand or functional electrical stimulation of paralyzed fingers. The most important challenge for current BMI design is to bring this technology more fully into the clinic. Thus, practical considerations, like understanding the differences in the neural representations of imagined and attempted movement (Vargas-Irwin et al., 2018) or force (Rastogi et al., 2020) by an individual with paralysis, are high priorities. In a similar vein, our results, suggesting that decoding the behavioral kinematic/kinetic mode from cortical activity is feasible, could increase the functionality of BMIs during object grasp. In addition, the improvement in behavioral mode decoding by using latent factors indicates that viewing the cortical motor control circuits as a dynamical system can facilitate the task of identifying cortical correlates of multiple behavioral modes. LFADS does not add information to that contained in the ECoG features, so its application may not always result in a large increase in decoding accuracy (especially in a discrete classification task; Fig. 6, S6), despite its effectiveness at uncovering low-dimensional representations (Fig. 4*B*,*D*, also from S6). However, the success of LFADS in improving decoding in some subjects, especially those with worse initial performance, suggests a potentially important role for denoising procedures such as LFADS in BMI future BMI applications. Improving decoding accuracy of behavioral mode from 77% to 91%, as in S4 (Fig. 6), would likely result in greatly improved overall BMI performance, more positive perceptions by the user, and better acceptance of the prosthesis.

The ubiquity of object-manipulation behaviors in human life underscores the importance of functioning hand grasp. In this case, however, ubiquity does not mean that the behavior is simple. The current study allowed us to examine the activity in human M1/PM that accompanied the sequential execution of movement and force. We found both movement and force to be quite well represented, allowing us to decode each with high accuracy. Our data also indicate that the movement and force representations are distinct, as we distinguished them in space, with LFADS, via the NVA, and via behavioral mode classification. The current results suggest that a BMI controlled using ECoG could restore both movement and isometric aspects of grasp to individuals with paralysis.

## Synthesis

Reviewing Editor: Mark Laubach, American University Decisions are customarily a result of the Reviewing Editor and the peer reviewers coming together and discussing their recommendations until a consensus is reached. When revisions are invited, a fact-based synthesis statement explaining their decision and outlining what is needed to prepare a revision will be listed below. The following reviewer(s) agreed to reveal their identity: Adam Rouse, Abidemi Ajiboye.

Your manuscript was reviewed by two experts and they both had significant issues with the manuscript as written. There is consensus that with extensive revisions, but not new experiments, all concerns can be addressed. Rather than providing an integrated synthesis over the two reviews, we felt it would be easiest to simply provide you with both reviews as they were written and to ask you to revise your paper to address all concerns that were raised. Please be sure to revise the reporting of all statistical tests and include reports of the statistical measure and degrees of freedom. Your paper seems relevant for use of estimation statistics as well. We look forward to your revised manuscript.

REVIEWER 1

This manuscript explores the spatiotemporal changes in ECoG signals during a one-finger index flexion task with both a movement phase before contact and isometric force phase after contact with a force sensor. The authors analyze the changes in spectral features across time on implanted microECoG arrays in 7 subjects. They identify clear spectral changes aligned to both movement onset and force sensor contact. Additionally, they show changes in the timing and pattern of activity across the grid of electrodes. When identifying latent factors with a dynamical systems model there are clear, repeatable neural trajectories even for single trials. I believe these results provide a nice and clear description of the complex spatiotemporal evolution of neural activity distributed across a wide brain area in humans for even a movement largely confined to a single degree of freedom. I think these are interesting findings but question whether they really address the central hypothesis presented of distinct representations of movement and force. Additionally, I have some reservations about the decoding results that are presented, and the metrics used. Overall, I think the experiment, dataset, and most of the analysis appear to be performed properly and exciting to the field but some of the interpretation should be refined and clarified.

My main concerns are:

1) I understand the challenges and limitations of designing experiments for human ECoG studies, but I feel the current experiment has significant challenges for claiming that movement and force representations in motor cortex are “distinct”. This is especially true in the title and Line 14 of the abstract. If I understand correctly, every trial consisted of a movement phase followed by a force phase. There were never any movement-only or force-only conditions. Additionally, while it appears there were instructed differences in force, no results are specifically given for the ability to decode these differences force. And it appears there was no instructed differences in the movement phase of the task. Thus, I think one should be careful in what one means by distinct movement and force representations. This study is a nice demonstration of the complex spatiotemporal evolution of neural activity but it is clear many channels and features are involved in both phases of movement and force. I think the use of the word “distinct” should be either better defended or likely removed for a different word choice.

2) In the discussion 479-490, it is said that low-frequency features modulated with movement onset while high-frequency features modulated with force onset yet we only have the example subjects in Figure 2. It seems like this is again picked up with the NVA and figure 5. It would be best if a summary statistics of low-frequency and high-frequency responses of Figure 2 was given. Or an alternative might be to more clearly link Figure 2 and Figure 5 along with how it is presented in the discussion so differences in timing of low and high-frequency responses can be more clearly evaluated.

3) Line 244 For the calculation of NVA, M^ref is the average 250 ms before the maximum force exertion. This seems like an unusual choice as it would seem to include part of the force production phase and would presumably make differences smaller as both the low and high frequency features go to near zero in the black maximum force window in Figure 5. It seems it would make since to either use a baseline around the cue or make the dataset zero-mean by subtracting the global average in some fashion. The author’s choice should be better explained, adjust the time window for M^ref, or perhaps rethink how the low-frequency and high-frequency feature clusters are being examined.

4) Lines 152-163. It’s unclear to me when the data for movement kinematics and force are being decoded. Is a continuous time decoder being built for the portion of the trial where just movement is happening or is the isometric portion included with presumably a lot of close to zero values. Similarly, when the finger is not in contact with the force transducer, is it a lot of zero values? What is being included could have large differences in what the FVAF means. It would be more impressive to have FVAF that was this high on only the movement or only the force phase versus all together which would likely include mainly a movement/force versus rest comparison. But conversely, combining altogether shows again the ability to detect the two phases. Either way, this should be clarified.

5) Differences are small for decoding between movement and force with either ECoG features or LFADS latent factors for S3, S5, S6, and S7. The differences in individual trial trajectories with LFADS compared to ECoG features for S5 and S6 are quite impressive. I’m therefore confused by these differences. It is perhaps a ceiling effect but the decoding isn’t that close to 1. Perhaps also, the temporal alignment isn’t as clean as it appears in Figure 4. It would be useful if there was more information for why this might be the case as this is important information to the reader for future data analysis and BCI applications. Or perhaps a running plot of movement phase and predicted movement phase to get a sense of how much of the error is offset in timing versus predicting the wrong class for the entire phase.

Other concerns:

-Line 140 - “We calculated the log-normalized spectral power in each ECoG electrode using short-time Fourier transforms (window width of 512 ms). We averaged the spectral power in 25-ms time bins.” Please clarify what averaged in 25-ms time bins means. Does that mean 25-ms time steps with a 512 ms window width?

-The time between movement and force production appear to be variable across trials and quite fast for certain trials as shown in Fig. 2. Was there any instruction to the subject to vary this and is there any other explanation for the variability, especially the very short time differences?

-Figure 2. The choice to align some data to movement onset and some to force onset is confusing and doesn’t allow for us to assess fairly whether high and low-frequency states are truly preferentially aligned to one or the other.

-Figure 3. The differences in effect sizes for Fig. 3B appear to be quite small. Part of that is the scaling but I also question whether FVAF for non-significantly tuned channels is adding a lot of noise to the effect. Additionally, I don’t see what subject this is in Fig. 1A and it includes 64 channels but parts of the manuscript suggest only using PM/M1 electrodes. All this should be clarified.

-There are no units for table 1

-Typo- line 8 - “accounted” rather than “account"

REVIEWER 2

Fundamentally, this is not a position vs force control task. If you look at Chib 2009, he had two specific tasks. In the position task, they had to perform a position tracking task while EXPLICITLY minimizing task (technically this itself is a hybrid task, whereas a true position only task would perform position control irrespective of force). Similarly, a force control task should only require the user to track force, as is correctly done in this experiment. But in your experiment there is no “position control”. There is just movement.

Another major challenge is that this is not a tightly controlled force task. The apparatus only measures normal force (well, I assume so, as the manuscript does not explicitly state this, but Figure 1B only reports 1 dimension of force). Off-axis forces (i.e. in the non-normal direction) producing similar normal forces would presumably have cortical differences, but this experimental paradigm would not decipher between the two. Form the Valero-Cuevas paper, “subjects wore a custom-molded thimble with a spherical Teflon bead embedded at its tip. This rigorously defined the mechanical task via a unique contact point and friction cone for force direction.” Your setup would allow the user to produce a force vector in different directions, and even a shear force

Related, there are a number of muscle patterns that could be used to produce the resultant task. You did not measure EMG activity related to the other fingers for stabilization. Presumably that activity was preceded by relevant cortical modulation, which would be a confounding factor in your recordings. For example, my guess is that if you measured FDI, EIP, and EDC activities, you would find trial-to-trial variations in the motor behavior.

It is not clear why after calculating the STFT using a 512ms window, you then averaged the spectral power in 25ms time bins

Why did you decide upon 10 bins of time history? How much time history was actually relevant? It seems that a more rigorous analysis could have elucidated the non-uniform time history of each feature.

If your k-means clustering was truly non-supervised, why did you specify 3 clusters? Might not the data have clustered into more clusters?

Your fundamental result, that low-frequency features did not different between force and movement, whereas high frequency features did, does not explain the data from Figure 2F. Why might in some cases low frequency features be involved in regulating force onset?

How does the result that low vs high frequency features differentially encode force onset relate to the current literature indicating that force is both cortically transient and tonic? Many studies suggest that force production is dominated by a transient phase cortically, where neural activity will actually return to baseline while constant force is maintained, but then changes activity with changing force requirements of the task (Maier et al. 1993; Hepp-Reymond and Kirkpatrick-Tanner 1999; Mason et al. 2002; Hendrix et al. 2009; Intveld et al. 2018). And, this transient phase is seen in both high and low frequency content. It is not clear to me that your results fit into this paradigm.

Mason C, Gomez J, Ebner T. Primary motor cortex neuronal discharg during reach-to-grasp:

controlling the hand as a unit. Archives Italiennes de Biologie. 2002;140(3):229 236.

Maier M, Bennett K, Hepp-Reymond M, Lemon R. Contribution of the monkey corticomotoneuronal system to the control of force in precision grip. J Neurophysiol. 1993;69(3):772-85.

Intveld RW, Dann B, Michaels JA, Scherberger H. Neural coding of intended and executed grasp force in macaque areas AIP, F5, and M1. Sci Rep-uk. 2018;8(1):17985.

Hepp-Reymond M, Kirkpatrick-Tanner M. Context-dependent force coding in motor and premotor cortical areas. Experimental Brain Research. 1999;128(128):123 133.

Hendrix CM, Mason CR, Ebner TJ. Signaling of grasp dimension and grasp force in dorsal premotor cortex and primary motor cortex neurons during reach to grasp in the monkey. J Neurophysiol. 2009;102(1):132 145.
